# Biochar and organic fertilizer drive the bacterial community to improve the productivity and quality of *Sophora tonkinensis* in cadmium-contaminated soil

**DOI:** 10.3389/fmicb.2023.1334338

**Published:** 2024-01-08

**Authors:** Han Liu, Cui Li, Yang Lin, Yi-jian Chen, Zhan-jiang Zhang, Kun-hua Wei, Ming Lei

**Affiliations:** ^1^National Center for TCM Inheritance and Innovation, Guangxi Botanical Garden of Medicinal Plants, Nanning, China; ^2^Guangxi Key Laboratory of Medicinal Resources Protection and Genetic Improvement, Guangxi Botanical Garden of Medicinal Plants, Nanning, China; ^3^Guangxi Engineering Research Center of TCM Resource Intelligent Creation, Guangxi Botanical Garden of Medicinal Plants, Nanning, China; ^4^The Third Affiliated Hospital of Heilongjiang University of Traditional Chinese Medicine, Harbin, China; ^5^Guangxi Key Laboratory for High-Quality Formation and Utilization of Dao-di Herbs, Guangxi Botanical Garden of Medicinal Plants, Nanning, China

**Keywords:** biochar and organic fertilizer, cadmium-contaminated soil, *Sophora tonkinensis*, bacterial community, yield and quality

## Abstract

Excessive Cd accumulation in soil reduces the production of numerous plants, such as *Sophora tonkinensis* Gagnep., which is an important and widely cultivated medicinal plant whose roots and rhizomes are used in traditional Chinese medicine. Applying a mixture of biochar and organic fertilizers improved the overall health of the Cd-contaminated soil and increased the yield and quality of *Sophora*. However, the underlying mechanism between this mixed fertilization and the improvement of the yield and quality of *Sophora* remains uncovered. This study investigated the effect of biochar and organic fertilizer application (BO, biochar to organic fertilizer ratio of 1:2) on the growth of *Sophora* cultivated in Cd-contaminated soil. BO significantly reduced the total Cd content (TCd) in the *Sophora* rhizosphere soil and increased the soil water content, overall soil nutrient levels, and enzyme activities in the soil. Additionally, the α diversity of the soil bacterial community had been significantly improved after BO treatment. Soil pH, total Cd content, total carbon content, and dissolved organic carbon were the main reasons for the fluctuation of the bacterial dominant species. Further investigation demonstrated that the abundance of variable microorganisms, including Acidobacteria, Proteobacteria, Bacteroidetes, Firmicutes, Chloroflexi, Gemmatimonadetes, Patescibacteria, Armatimonadetes, Subgroups_ 6, *Bacillus* and *Bacillus_ Acidiceler*, was also significantly changed in Cd-contaminated soil. All these alterations could contribute to the reduction of the Cd content and, thus, the increase of the biomass and the content of the main secondary metabolites (matrine and oxymatrine) in *Sophora*. Our research demonstrated that the co-application of biochar and organic fertilizer has the potential to enhance soil health and increase the productivity and quality of plants by regulating the microorganisms in Cd-contaminated soil.

## 1 Introduction

Cadmium (Cd) is a toxic heavy metal widely present at low concentrations in natural environments (Li et al., 2012; Ming et al., 2018). In recent decades, the contamination of agricultural soils with Cd has escalated due to various factors, such as mining and other industrial activities, phosphate fertilization, fossil fuel combustion, and so on (Todd et al., 2018; Wei and Yan, 2023). Even though Cd content in the soil is very low, continuous low levels of Cd exposure can also lead to excessive Cd accumulation in plants (Wei and Yan, 2023). Most importantly, this non-degradable heavy metal gets transmitted to humans along the food chain (Nagajyoti et al., 2010; Wu et al., 2019; Bai et al., 2023). Even at minimal levels of exposure, Cd can induce kidney and skeletal diseases, cardiac metabolic dysfunction, cancer, and mortality in humans (Menahem et al., 2018; Hui et al., 2019; Rachael et al., 2019). Additionally, excessive Cd accumulation in the soil can disrupt the metabolic processes of plants (Meghan et al., 2017; Zhu et al., 2020), such as protein synthesis (Lian et al., 2015; Sha et al., 2019), nitrogen and carbohydrate metabolism (Huang et al., 2020; Zhu et al., 2022; Cao et al., 2023), enzyme activation (Chmielowska and Deckert, 2012; Lian et al., 2018; AlHuqail et al., 2023), photosynthesis, and chlorophyll synthesis (Abeer et al., 2016; Raletsena et al., 2022; Tunçtürk et al., 2023), leading to abnormal plant growth and metabolism and ultimately mortality (Mohammadhossein et al., 2019; Li et al., 2023). Therefore, reducing Cd concentrations in agricultural soils is crucial for sustainable agriculture and human health.

*Sophora tonkinensis* Gagnep. is a leguminous shrub widely distributed in China, South Korea, and Vietnam and is well-known for its commercial and medicinal applications (Hao et al., 2019). In China, the desiccated roots and rhizomes of *Sophora* are commonly known as “Shan-Dou-Gen” and have been included in the Chinese Pharmacopoeia (Ying et al., 2020). *Sophora* has been used in traditional Chinese medicine (TCM) to treat various ailments, including tumors, abdominal pain, fever, throat inflammation, asthma, dermatitis, and gastrointestinal bleeding (Rui et al., 2020; Nan et al., 2021; Xia et al., 2022). However, the wild reserves of *Sophora* are currently at risk due to excessive harvesting driven by growing market demand and habitat deterioration in natural areas. Thus, artificial cultivation has emerged as the primary means of sourcing *Sophora* for medicinal purposes; however, it is still unable to meet market demand (Shaza et al., 2019). Moreover, *Sophora* fields are frequently contaminated with Cd, which reduces soil health and increases Cd accumulation in plants.

Biochar is an inexpensive and effective physical material for contaminated soil remediation. Biochar is a solid material produced through high-temperature biomass pyrolysis in an environment devoid of oxygen (Yang L. et al., 2020; Yang Y. W. et al., 2020). It has a high carbon content and is frequently employed for remediating soil contaminated with heavy metals due to its complementary functional groups, high specific surface area, and high cation exchange capacity (Tan et al., 2015; Li et al., 2017; Sheng et al., 2020; Yang L. et al., 2020; Yang Y. W. et al., 2020; Li et al., 2022). Biochar can also increase soil enzyme and bacterial activities (Muhammad et al., 2018; Effrey et al., 2019; Shen et al., 2019; Wan et al., 2023; Xiao et al., 2023). However, long-term single-use of biochar can also have drawbacks, such as reducing the effectiveness of plant nitrogen (Bo et al., 2023; Wang et al., 2023; Zhang C. et al., 2023; Zhang N. H. et al., 2023; Zhang X. et al., 2023; Zhang Z. et al., 2023; Zheng K. et al., 2023; Li et al., 2024). In practice, biochar is commonly used with supplemental nutrients, such as organic fertilizer, to enhance soil quality, mitigate heavy metal stress, and increase crop productivity (Muhammad et al., 2017; Saeed et al., 2021). Notably, this fertilization method is often accompanied by changes in the soil bacterial community (Wei et al., 2020; Liu et al., 2022; Wen et al., 2023). However, to date, the alterations and effects of the soil bacterial community in most soil remediation processes are still poorly understood.

In the present study, we investigate the effects of a mixture of biochar and organic fertilizer applications on *Sophora* rhizosphere soil quality, *Sophora* growth and Cd accumulation, and the *Sophora* rhizosphere bacterial community by conducting a field-controlled experiment. We analyzed the correlation between microbial species changes and soil physicochemical factors and between species and *Sophora* yield and quality. Our study will not only help to reduce Cd contamination on soil and plants but also have positive implications in explaining the mechanism of soil remediation.

## 2 Materials and methods

### 2.1 Study site

The experiment was conducted in a *Sophora tonkinensis* field in Jinchengjiang District, Hechi City, Guangxi Zhuang Autonomous Region, China (108°001 E, 24°427 N, altitude 207 m). The site is adjacent to a non-ferrous metal smeltery and was contaminated with Cd. The area has a subtropical monsoon climate, with an average sunshine of 1350.9 h, average precipitation of 3541.36 mm, and a soil pH of 5.52. The majority of rainfall occurs between May and August, while the average high and low temperatures are 26°C and 18°C, respectively.

### 2.2 Preparation of a biochar and organic fertilizer mixture

Biochar (carbonized rice straw) was purchased (Shanghai Puzhi Environmental Technology Co., Ltd., China). It was prepared by continuously carbonizing rice straw at a pyrolysis temperature of 400°C for 30 min. The biochar has a microporous specific surface area of 62.637 m^2^/g, a pore size of 5.012 nm, a pore capacity of 0.076 cm^3^/g, total nitrogen of 0.76%, total carbon of 22.64%, total hydrogen of 1.18%, total oxygen of 10.46%, and total sulfur of 0.25%. The organic fertilizer used in the experiment was chicken manure (purchased from Hebei Dewuoduo Fertilizer Co., Ltd., China). It has an organic matter content of 60%, total nitrogen content of 1.6%, a phosphorus pentoxide (P_2_O_5_) content of 4.5%, a potassium oxide (K_2_O) content of 3%, and a pH of 6.6. The soil amendment required for the experiment was prepared by thoroughly mixing biochar and organic fertilizer at a ratio of 1:2.

### 2.3 Experimental design and implementation

In the present study, the soil contaminated with the heavy metal Cd is referred to as the HM group; the fields cultivated with *Sophora* in the HM are referred to as the FS group; and a combination of biochar and organic fertilizer application at a ratio of 1:2 on the *Sophora* fields in the Cd-contaminated soil is referred to as the BO group. The experimental period lasted for 14 months. In the FS treatment, *Sophora* was sown in March 2021 on an area of approximately 0.02 ha, maintaining a planting density of 64,800 plants/ha, a row spacing of 45 cm, and a plant spacing of 40 cm; the harvesting was done in May 2022. The other field management measures, except for fertilization, were the same as the traditional farming methods in the area. The measures in the BO treatment were the same as those in the FS treatment. Besides, in BO, a mixture of biochar and organic fertilizer was applied as a base fertilizer before planting *Sophora* at a fertilization rate of 420 kg/ha; 147 kg/ha of the fertilizer was applied as topdressing in November 2021.

### 2.4 Soil and plant sampling

During the harvesting period in May 2022, a random sampling method was used to collect the soil samples from each sampling site. First, the topsoil at a depth of 0 ~ 5 cm was removed with a shovel. Then, the soil sample for the HM treatment was collected from the layer 5 ~ 20 cm below the soil surface. The whole *Sophora* plant was dug out of the soil in the FS and BO treatments without damaging the roots. The soil blocks loosely attached to the roots was shaked off and discarded, and then the soil tightly attached to the roots was collected using a brush. After drying, the soil was used to measure its physical and chemical properties. The *Sophora* plants were then placed in a bag and taken to the laboratory. The roots, stems, and leaves were separated, dried in an oven at a constant temperature, and crushed with a grinder to obtain a fine powder; this powder was then passed through a 0.15 mm sieve. The remaining soil from the roots of *Sophora* was also collected and divided into two parts; one was stored in a− 80°C freezer for measuring enzyme activity, and the other was stored in a− 80°C freezer for DNA extraction.

### 2.5 Analysis of soil physicochemical properties, plant properties, and Cd content

Soil pH was measured using a pH meter (Reze PHS-3E pH measuring instrument, China), and soil total carbon content was measured using an element analyzer (Elemental Vario EL Cube, Germany). Soil organic matter content was measured using a potassium dichromate volumetric method, soil alkaline nitrogen content was measured using an alkaline hydrolysis diffusion method, soil available potassium content was measured using an ammonium acetate extraction flame photometric method (Liu et al., 2021), soil Cd content was measured using an inductively coupled plasma mass spectrometry analyzer (Muhammad et al., 2018), and soil bulk density and water content were measured using a cutting ring method (Sun et al., 2022). Soil enzyme activity was measured using the method by İbrahim and Md (İbrahim et al., 2020; Md et al., 2020). The Cd content of plants was determined by inductively coupled plasma mass spectrometry, while matrine and oxymatrine content was measured by the HPLC method (Thang et al., 2022).

### 2.6 Analysis of soil bacterial diversity

Bacterial genomic DNA was extracted from the soil samples using the HiPure Soil DNA Kit (Magen, China), and the V3-V4 region was amplified by PCR using bacterial primers 341F (5′-CCTACGGGNGGCGWGCAG-3′) and 806R (5′-GACTACHVGGGTATCTAAT-3′). PCR was carried out in a 50-μL reaction volume using TransGen High-Fidelity PCR SuperMix (TransGen Biotech, China), forward and reverse primers (0.2 μmol/L), and template DNA (5 ng). The obtained amplicons were evaluated on 2% agarose gels and purified using the AxyPrep DNA Gel Extraction Kit (Axygen Biosciences, United States) according to the manufacturer’s instructions. Further, sequencing libraries were generated from these amplicons using the SMRTbell TM Template Prep Kit (PacBio, United States). Library quality was assessed with a Qubit 3.0 fluorometer (Thermo Fisher Scientific, United States) and a FEMTO pulse system (Agilent Technologies, United States). Finally, the libraries were sequenced on the PacBio Sequel platform. The raw reads were deposited in the NCBI Sequence Read Archive (SRA) database (accession number: PRJNA1025955).

In addition, the raw reads were quality filtered by removing the reads containing more than 10% unknown nucleotides (N) and reads containing less than 50% bases with a mass (Q value) greater than 20 using FASTP (version 0.18.0) (Chen et al., 2018). The paired-end clean reads were merged as raw tags using FLASH (version 1.2.11) (Magoč and Salzberg, 2011) with a minimum overlap of 10 bp and a mismatch error rate of 2%. Quality filtering of the raw tags was performed under specific filtering conditions to obtain high-quality, clean tags. The filtering conditions were set as follows: (1) the raw tags from the first low-quality base site where the number of bases in the continuous low-quality value (the default quality threshold is ≤3) reached the set length (the default length is 3 bp) were broken; (2) then, the tags whose continuous high-quality base length was less than 75% of the tag length were filtered. The clean tags were clustered into operational taxonomic units (OTUs) with ≥97% similarity using the UPARSE pipeline (version 9.2.64) (Edgar, 2013). All chimeric tags were removed using the UCHIME algorithm (Edgar, 2013), and the obtained effective tags were used for further analysis. The tag sequence with the highest abundance was selected as the representative sequence within each cluster. Then, paired-end denoised reads were merged as raw ASVs (amplicon sequence variants) with a minimum overlap of 12 bp. Chimera sequences were identified and deleted using the UCHIME algorithm (Edgar, 2011). After chimera removal, the denoised, chimera-free ASV sequences and their abundances were output.

### 2.7 Statistical analysis

Excel 2019 and IBM SPSS Statistics 22 were used to analyze the experimental data, and Excel 2019 was used to plot the graphs. One-way analysis of variance (ANOVA), two-tailed Duncan’s test, and Tukey’s HSD test (*p* < 0.05) were used to evaluate the significant differences between treatment means. The Shannon, Simpson, and Pielou’s evenness indexes were calculated in QIIME (version 1.9.1) (Wang, 2007; Caporaso, 2010; Nilsson et al., 2018). The alpha-diversity indices of the different treatments were compared by performing Tukey’s HSD test in the R package Vegan (version 2.5.3) (Vegan, Community Ecology Package, 2010). Principal component analysis (PCA) was performed using the R package Vegan (Vegan, Community Ecology Package, 2010). Statistical analysis of the Adonis test (based on PERMANOVA) was calculated in the R package Vegan (Vegan, Community Ecology Package, 2010). Venn analysis was performed using the Venn diagram package in R (version 1.6.16). Species between groups were compared by multiple comparisons based on Tukey’s HSD in the R package Vegan (Vegan, Community Ecology Package, 2010). Biomarker features in each group were screened using LEfSe software (version 1.0) (Caporaso, 2010). Redundancy analysis (RDA) and the Envfit test were executed in the Vegan package (Vegan, Community Ecology Package, 2010) to clarify the influence of environmental factors on community composition. The Pearson correlation coefficient between environmental factors and species was calculated using the Psych package in R (version 1.8.4) (The Comprehensive R Archive Network, 2015). A network based on the Pearson correlation coefficient was generated using Omicsmart, a dynamic, real-time interactive online platform for data analysis.^1^

## 3 Results

### 3.1 Soil physicochemical properties

**Figure 1:**
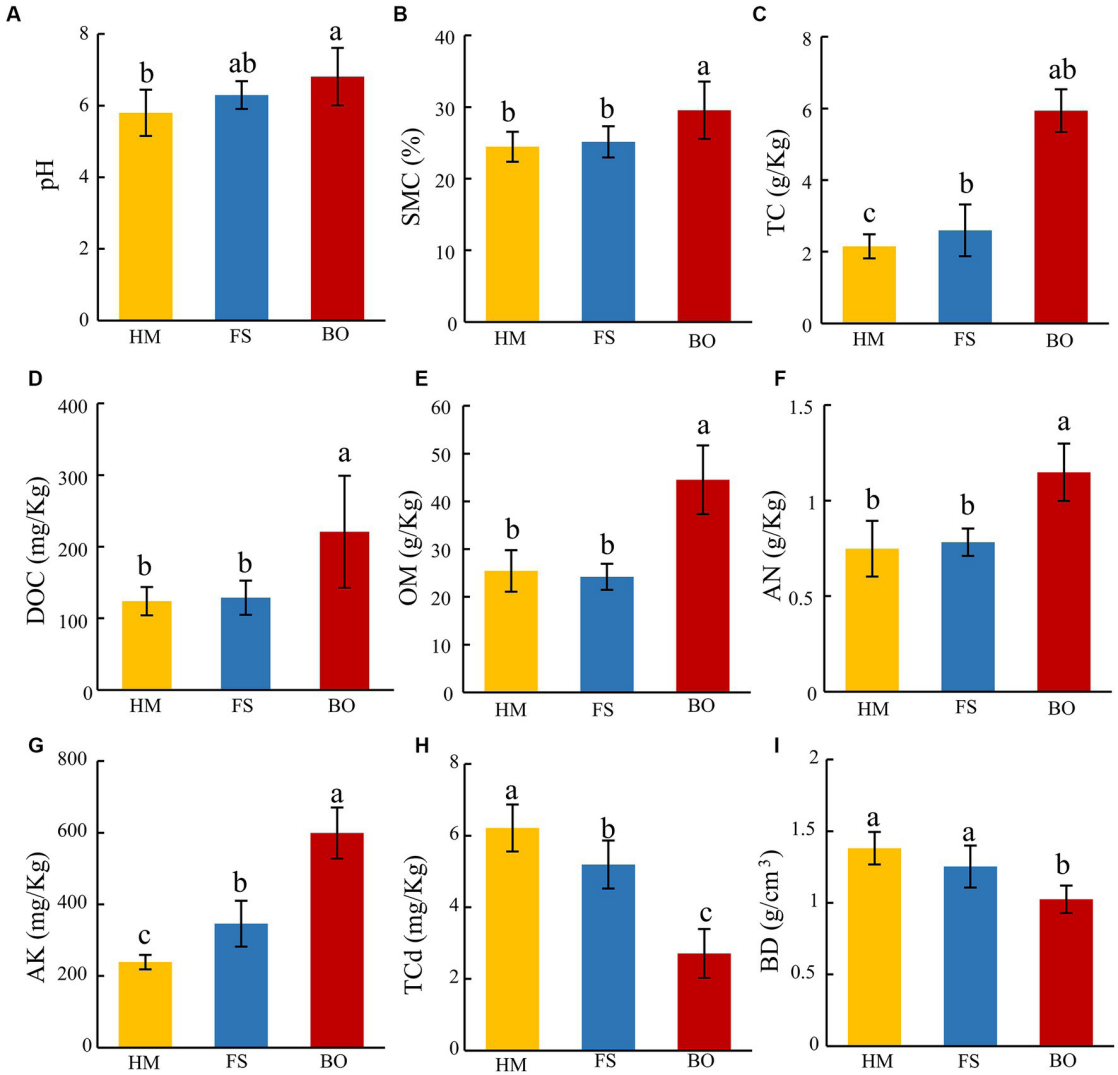
Soil physicochemical properties. **(A)** soil pH; **(B)** soil moisture content (SMC); **(C)** soil total carbon content (TC); **(D)** soil dissolved organic carbon content (DOC); **(E)** soil organic matter content (OM); **(F)** soil alkali-N content (AN); **(G)** soil available potassium (AK); **(H)** soil total cadmium content (TCd); **(I)** soil bulk density content BD. Data are presented as mean ± standard deviation, *n* = 6. Different lowercase letters indicate significant differences among treatments (ANOVA); differences were considered significant at *p* < 0.05.

We first evaluated the physical and chemical characteristics of the soils after the application of the biochar and organic fertilizer mixture. Compared with FS, BO treatment resulted in a significant increase in pH, soil moisture content (SMC), total carbon content (TC), dissolved organic carbon content (DOC), organic matter content (OM), soil alkali-N content (AN), and available potassium content (AK) (17.42, 20.81, 183.88, 78.61, 75.04, 53.42, 150.77%, respectively; Figures 1A–G). Compared to FS, BO treatment also resulted in a significant increase in SMC, TC, DOC, OM, AN, and AK (17.54, 88.94, 71.44, 83.91, 46.79, and 73.17%, respectively, Figures 1B–G). In addition, BO treatment significantly reduced soil total Cd content (TCd, 56.37%) and soil bulk density (BD, 25.79%) (Figures 1H,I). The cultivation of *Sophora* plants also partially remediated soil Cd contamination. Compared to HM, the FS treatment decreased soil TCd by 16.42% and increased TC and AK by 50.25 and 44.81%, respectively (Figures 1H,I). All the differences observed in this experiment were statistically significant (*p* < 0.05; Figure 1).

### 3.2 Soil enzyme activity

To gain additional insight into the effects of biochar and organic fertilizer mixture applications on soil health, we assessed the soil enzyme activity. Compared with HM, BO treatment increased the activity of catalase, polyphenol oxidase, and β-glucosidase by 50.75, 68.11, and 233.94%, respectively (Table 1). Similarly, compared with FS, BO treatment increased catalase activity, polyphenol oxidase activity, and β-glucosidase activity by 41.24, 50.37, and 207.41%, respectively (Table 1). All the differences observed in this experiment were statistically significant (*p* < 0.05). However, there was no significant difference in cellulase activity among the three groups.

**Table 1:** Soil enzyme activity in *Sophora* fields under different treatments.

Treatments	Catalase(mmoL/d/g)	Polyphenol oxidase (μmoL/h/g)	β-Glucosidase (μmoL/d/g)	Cellulase (mg/d/g)
HM	41.79 ± 4.50^b^	0.32 ± 0.05^b^	5.23 ± 2.53^b^	18.44 ± 2.51^ns^
FS	44.61 ± 6.67^b^	0.36 ± 0.03^b^	5.68 ± 0.89^b^	18.73 ± 4.16^ns^
BO	63.01 ± 8.80^a^	0.54 ± 0.14^a^	17.45 ± 1.68^a^	24.43 ± 5.73

All data are presented as mean ± standard deviation. Different letters in the same column indicate significant differences (ANOVA) between treatments at *p* < 0.05; ns, *p* ≥ 0.05 (*n* = 6).

### 3.3 Growth parameters and Cd content of *Sophora*

Analysis of *Sophora* growth parameters revealed that the mixed application of biochar and organic fertilizer increased *Sophora* main root length, aboveground dry biomass, and dry root biomass by 49.02%, 34.23%, and 28.78%, respectively, but decreased plant desiccation rate by 8.86% (Table 2). All the differences observed in this experiment were statistically significant (*p* < 0.05; Table 2).

**Table 2:** Growth parameters and drying rate of *Sophora* under different treatments.

Treatments	Main root length (cm)	Aboveground dry biomass (g)	Dry root biomass (g)	Plant drying rate (%)
FS	15.30 ± 3.70^*^	10.15 ± 1.66^*^	6.05 ± 1.50^*^	30.59 ± 3.63^ns^
BO	22.80 ± 4.70	13.47 ± 2.52	7.79 ± 0.95	27.88 ± 4.49

*, *p* < 0.05; ns, *p* ≥ 0.05 (ANOVA) (*n* = 6).

The study also investigated the effect of mixed fertilization on the Cd accumulation in different parts of *Sophora*. Compared with FS, BO treatment decreased the Cd content in the roots, stems, and leaves by 32.35%, 27.97%, and 35.64%, respectively (*p* < 0.05; Table 3).

**Table 3:** Effect of biochar and organic fertilizer combination on the cadmium content in the roots, stems, and leaves of *Sophora.*

Treatments	Cd content (μg/g)		
	Root	Stem	Leaves
FS	568.75 ± 83.24*	153.32 ± 45.2*	140.23 ± 35.05*
BO	384.79 ± 79.83	110.44 ± 34.06	90.25 ± 18.76

*, *p* < 0.05; ns, *p* ≥ 0.05 (ANOVA) (*n* = 6).

### 3.4 Matrine and oxymatrine content of *Sophora*

**Figure 2:**
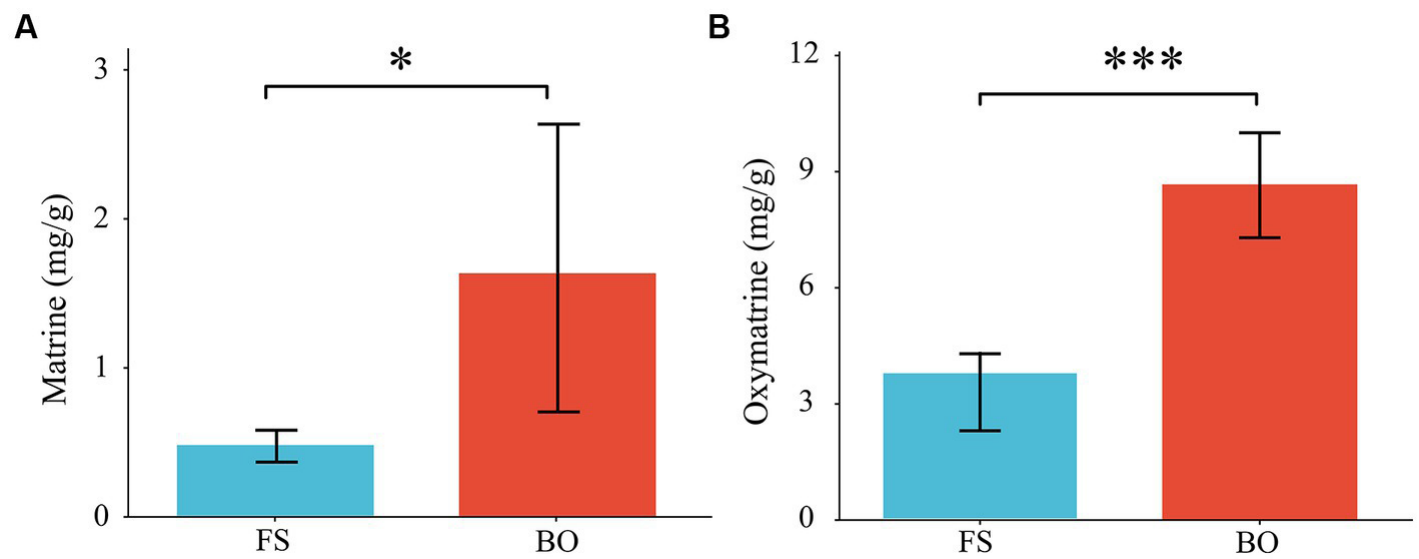
Matrine and oxymatrine content of *Sophora*. Data are presented as mean ± standard deviation. **(A)** matrine content; **(B)** oxymatrine content. Different lowercase letters indicate significant differences between treatments (ANOVA) *p < 0.05; **p < 0.01; ***p < 0.001, *n* = 6.

We also determined the content of matrine and oxymatrine in *Sophora*. Compared with those of FS, the levels of matrine and oxymatrine in *Sophora* under BO treatment increased by 216.13% and 108.83%, respectively (*p* < 0.05; Figure 2).

### 3.5 Changes in α-diversity of soil bacteria under different treatments

**Figure 3:**
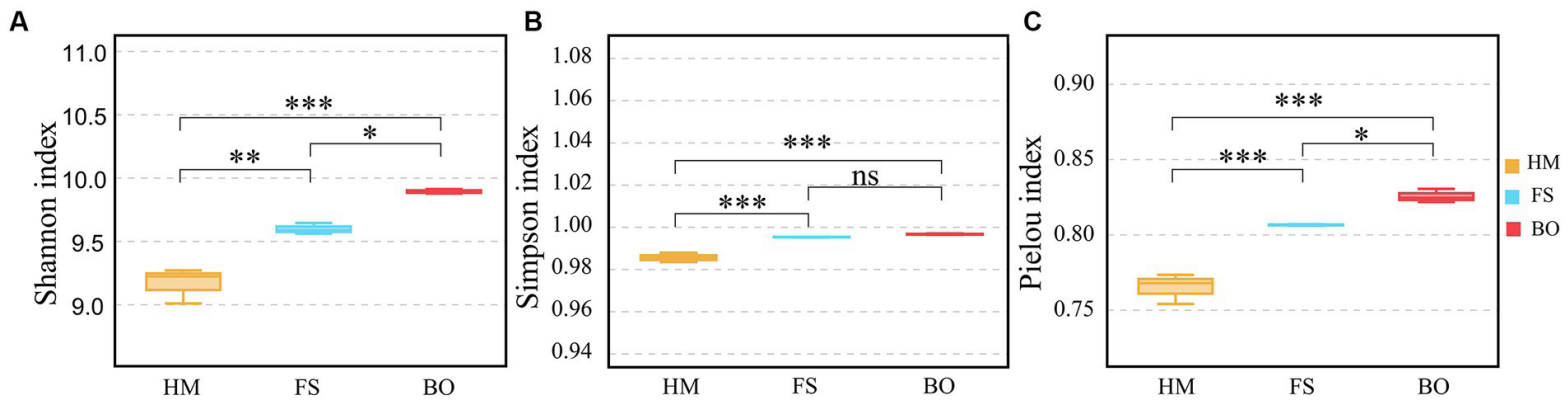
Alpha diversity indices of bacterial communities. **(A)** Shannon index; **(B)** Simpson index; **(C)** Pielou index. All these indices were calculated and compared at the OTU level. Note: Data are presented as mean ± standard deviation (Tukey HSD), *p < 0.05; ***p < 0.001, ns, *p* ≥ 0.05.

The study investigated the α-diversity of soil bacteria in Cd-contaminated soil after BO treatment. We analyzed the Shannon, Simpson, and Pielou indices to evaluate the diversity of the bacterial community. The results indicated that the Shannon index and Pielou index of soil bacteria in the BO group were significantly higher (*p* < 0.05; 3.08 and 2.37%, respectively) than those in the FS group (Figures 3A,C). However, no significant difference in the Simpson index was observed between the two groups (Figure 3B). Compared with HM, the FS treatment increased the Shannon, Simpson, and Pielou indices by 4.70, 0.98%, and 5.41, respectively (*p* < 0.05; Figures 3A–C). Meanwhile, the Shannon, Simpson, and Pielou indices of the BO group were 7.92%, 1.12%, and 7.91% higher than those of the HM group, respectively, and the differences were significant (*p* < 0.05; Figure 3).

### 3.6 Changes in β-diversity of soil bacteria under different treatments

**Figure 4:**
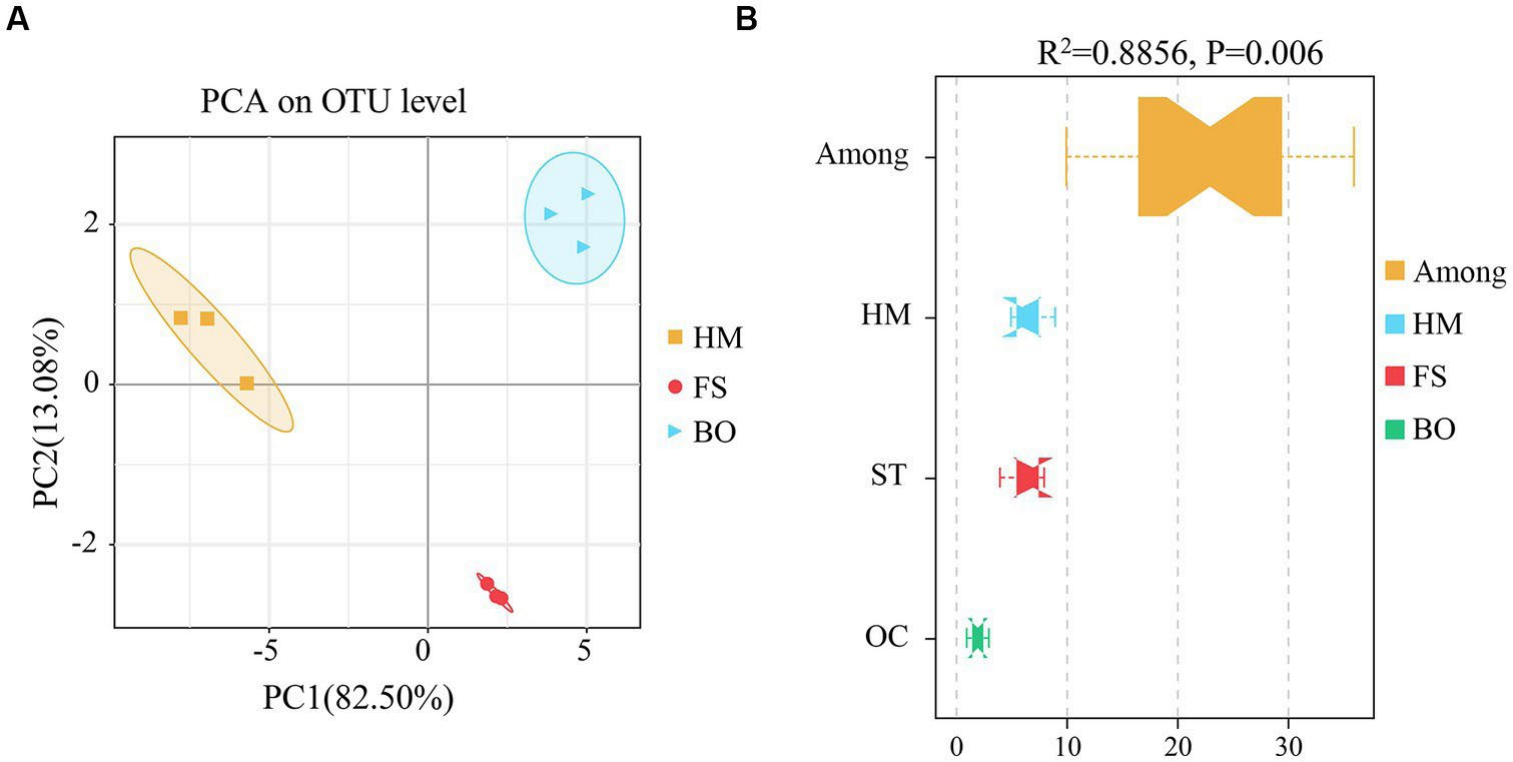
β-diversity of soil bacteria under different treatments. **(A)** PCA based on the weighted Unifrac distance at the OTU level (PCA, Principal Component Analysis); **(B)** Adonis analysis based on the weighted Unifrac distance at the OTU level (permutational multivariate analysis of variance, PERMANOVA).

Furthermore, both PCA and Adonis analyses based on the weighted Unifrac distance at the OTU level were performed to examine the overall structural differences in the soil bacterial community under different treatments. PCA divided the bacterial communities into three groups (HM, FS, and BO) (Figure 4A). Compared with HM, the Adonis test (PERMANOVA) showed significant alterations in the structure of the soil bacterial community under FS and BO treatments (*R*^2^ = 0.8856, *p* = 0.006) (Figure 4B). In addition, the dissimilarities between the HM, FS, and BO groups were more pronounced than the dissimilarities within each group (Figure 4B).

### 3.7 Analysis of bacterial community composition

**Figure 5:**
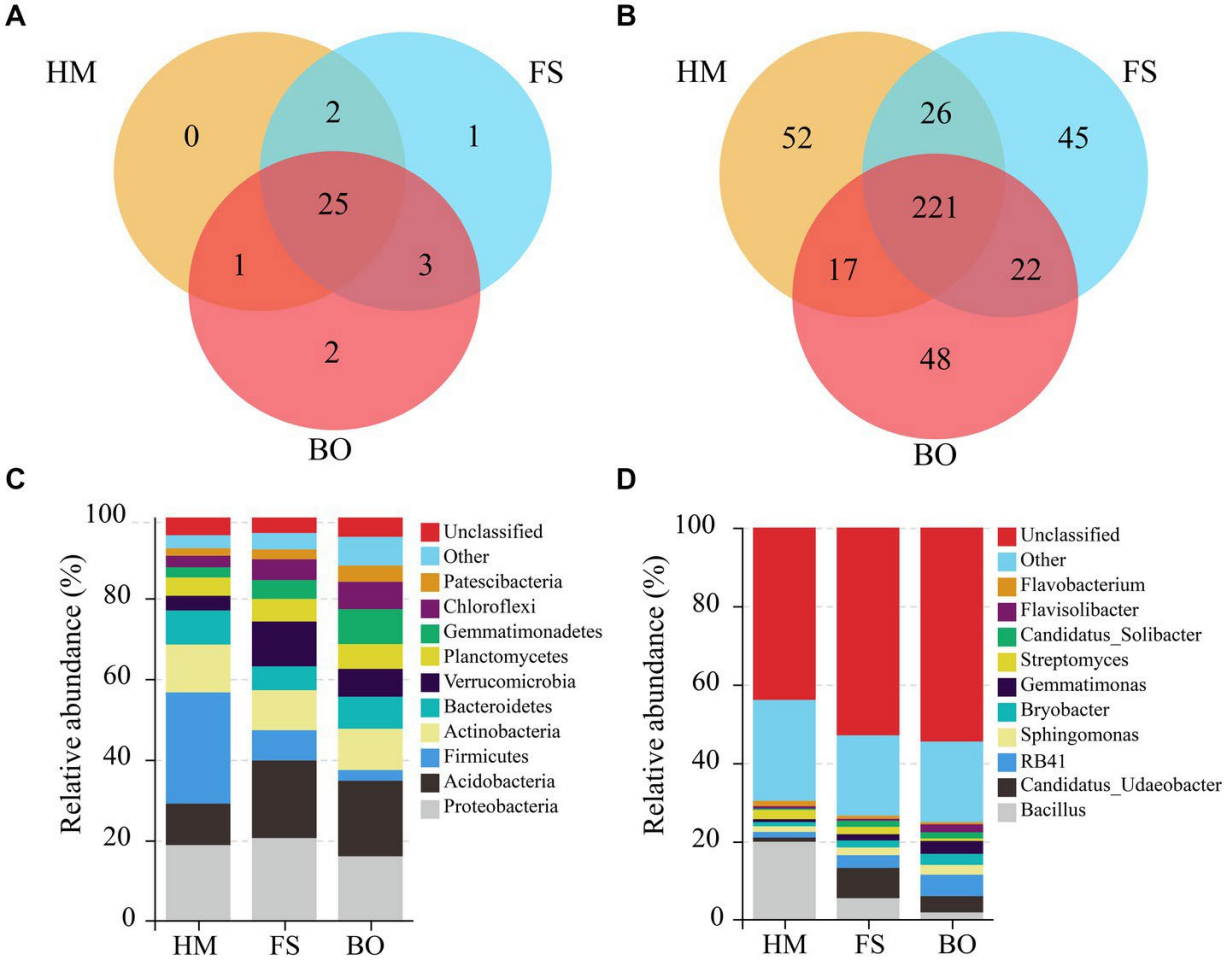
Venn diagram based on the phyla **(A)** and genera **(B)** of the soil bacteria in the rhizospheres of the HM, FS, and BO treatments. The relative abundance of the dominant bacterial phylum **(C)** and bacterial genus **(D)** in the *Sophora* rhizosphere under different treatments.

In order to understand the impact of BO treatment on the species distribution of the bacterial community, further analysis was conducted. As shown in Figure 5A, there were 25 identical soil bacterial phyla among the HM, FS, and BO groups. The FS group had one distinct phylum, the BO group had two unique phyla, and the HM group had no unique bacterial phyla. Furthermore, 221 identical soil bacterial genera were observed among the three groups (Figure 5B). In total, 52, 45, and 48 unique genera were found in the HM, FS, and BO, respectively.

Analysis based on species distribution revealed that the soil bacterial community encompassed 34 phyla, 274 families, and 428 genera. The top ten dominant bacterial phyla, ranked by relative abundance, were Proteobacteria (18.46%), Acidobacteria (16.08%), Firmicutes (12.58%), Actinobacteria (10.66%), Bacteroidetes (7.44%), Verrucomicrobia (7.20%), Chloroflexi (4.93%), Planctomycetes (5.44%), Gemmatimonadetes (5.28%), and Patescibacteria (2.78%). Collectively, these bacterial phyla accounted for over 90% of all sequences (Figure 5C). Similarly, the top ten dominant genera, based on the relative abundance, were Bacillus (9.11%), *Candidatus Udaeobater* (4.31%), *RB41* (3.38%), *Sphingomonas* (1.96%), *Gemmatimonas* (1.88%), *Bryobater* (1.91%), *Streptomyces* (1.61%), *Flavobacterium* (0.92%), *Paenibacillus* (0.83%), and *Flaviobacterium* (1.05%); these bacterial genera account for more than 26.96% of all sequences (Figure 5D).

Among the aforementioned bacterial phyla, compared to FS, BO treatment resulted in a substantial increase in the relative abundance of Bacteroidetes, Chloroflexi, and Patescibacteria in the rhizosphere soil of *Sophora*. Conversely, a significant decrease in the relative abundance of Proteobacteria, Firmicutes, and Verrucomicrobia was detected in the rhizosphere soil. Compared to HM, the FS treatment increased the relative abundance of Verrucomicrobia, Chloroflexi, and Gemmatimonadetes, but decreased that of Firmicutes and Bacteroidetes. In comparison to HM, BO treatment significantly increased the relative abundance of Acidobacteria, Verrucomicrobia, Chloroflexi, Planctomycetes, Gemmatimonadetes, and Patescibacteria but decreased that of Proteobacteria and Firmicutes (Supplementary Table S1). Among the aforementioned bacterial genera, compared to FS, BO treatment resulted in a significant increase in the relative abundance of *RB41*, *Flavisolibacter*, *Gemmatimonas*, *Bryobacter*, and *Sphingomonas* in the rhizosphere soil of *Sophora*. Conversely, a significant decrease in the relative abundance of *Flavobacterium*, *Streptomyces*, *Candidatus_Udaeobacter*, and *Bacillus* was detected in the rhizosphere soil. Compared to HM, the FS treatment significantly increased the relative abundance of *Candidatus_Udaeobacter*, *RB41*, *Sphingomonas*, *Bryobacter*, and *Candidatus_Solibacter*, but significantly reduced that of *Bacillus*, *Gemmatimonas*, and *Flavobacterium*. In comparison to HM, BO treatment significantly increased the relative abundance of *Candidatus_Udaeobacter*, *RB41*, *Sphingomonas*, *Bryobacter*, *Gemmatimonas*, *Candidatus_Solibacter*, and *Flavisolibacter*, but significantly decreased that of *Bacillus*, *Streptomyces*, and *Flavobacterium* (Supplementary Table S2).

**Figure 6:**
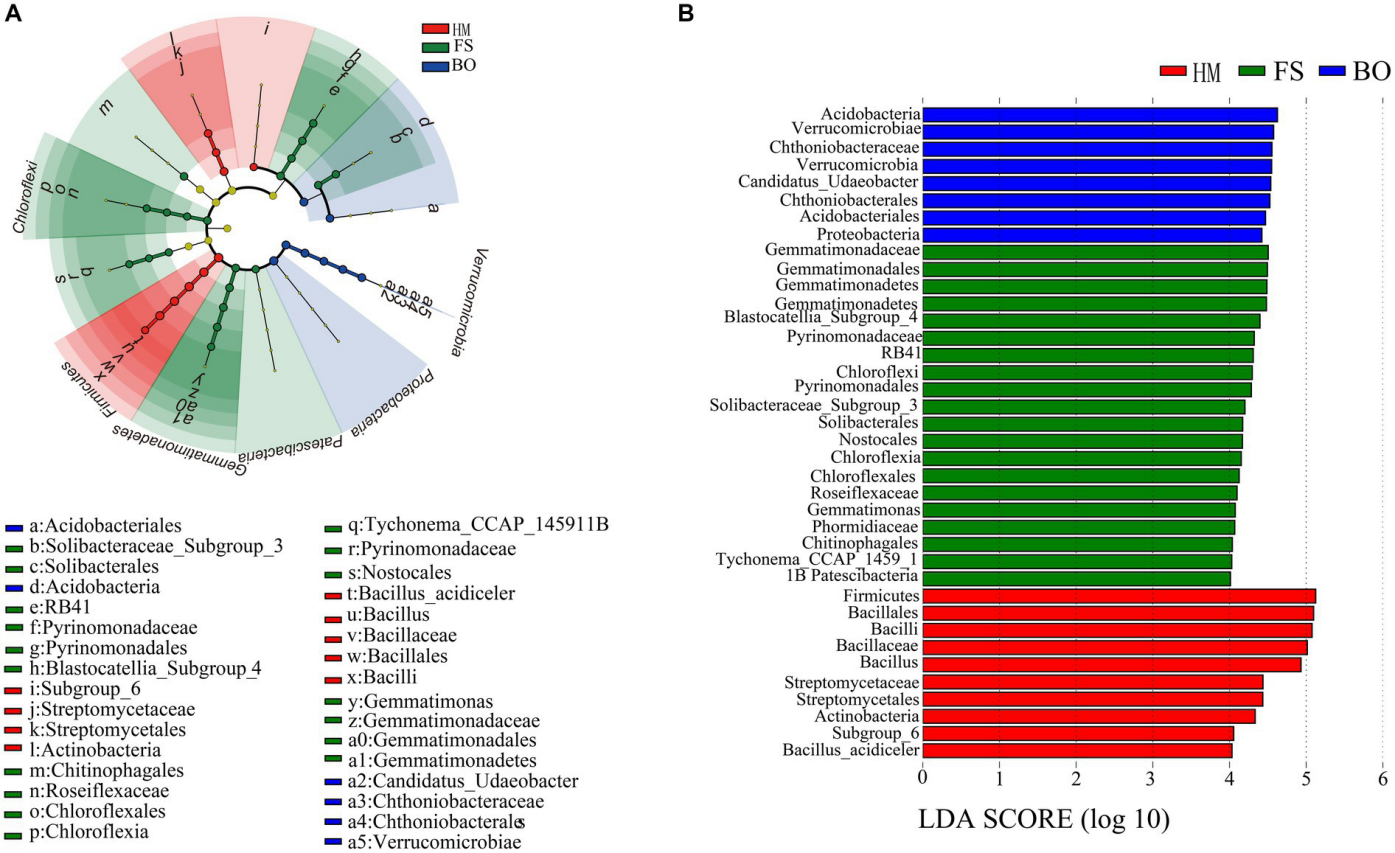
LEfSe analysis of soil bacteria in *Sophora* fields. **(A)** Cladogram diagram of bacterial lineages in *Sophora* rhizosphere soil; **(B)** indicator soil bacteria with LDA scores of 4.0 from in *Sophora* rhizosphere soil.

LEfSe analysis was performed to determine the differences in soil bacteria among the three groups. Firmicutes phylum, Subgroup_6 class, Bacilli class, Actinobacteria class, Streptomycetales order, Bacillales order, Bacillaceae family, Streptomycetaceae family, *Bacillus* genus, and *Bacillus_acidiceler* species were significantly enriched in the HM group (Figure 6A). Verrucomicrobia phylum, Proteobacteria phylum, Verrucomicrobiae class, Acidobacteriia class, Chthoniobacterales order, Acidobacteriaes order, Chthoniobacteraceae family, and *Candidatus_Udaeobacter* genus were significantly enriched in the FS group (Figure 6A). In addition, 20 dominant species were significantly enriched in the BO group, including Gemmatimonadetes phylum, Chloroflexi phylum, Patescibacteria phylum, Blastocatellia_Subgroup_4 class, Gemmatimonadetes class, Chloroflexia class, Pyrinomonadales order, Nostocales order, Solibacterales order, Gemmatimonadales order, Chloroflexales order, Chitinophagales order, Pyrinomonadaceae family, Solibacteraceae_Subgroup_3 family, Gemmatimonadaceae family, Roseiflexaceae family, Phormidiaceae family, *Tychonema_CCAP_1459_11B* genus, *Pyrinomonadaceae*, *RB41* genus, and *Gemmatimonas* genus (Figure 6A). In total, 38 evolutionary branches of soil bacteria showed significant differences (LDA > 4) (Figure 6B).

### 3.8 Relationships between bacterial communities and environmental factors

**Figure 7:**
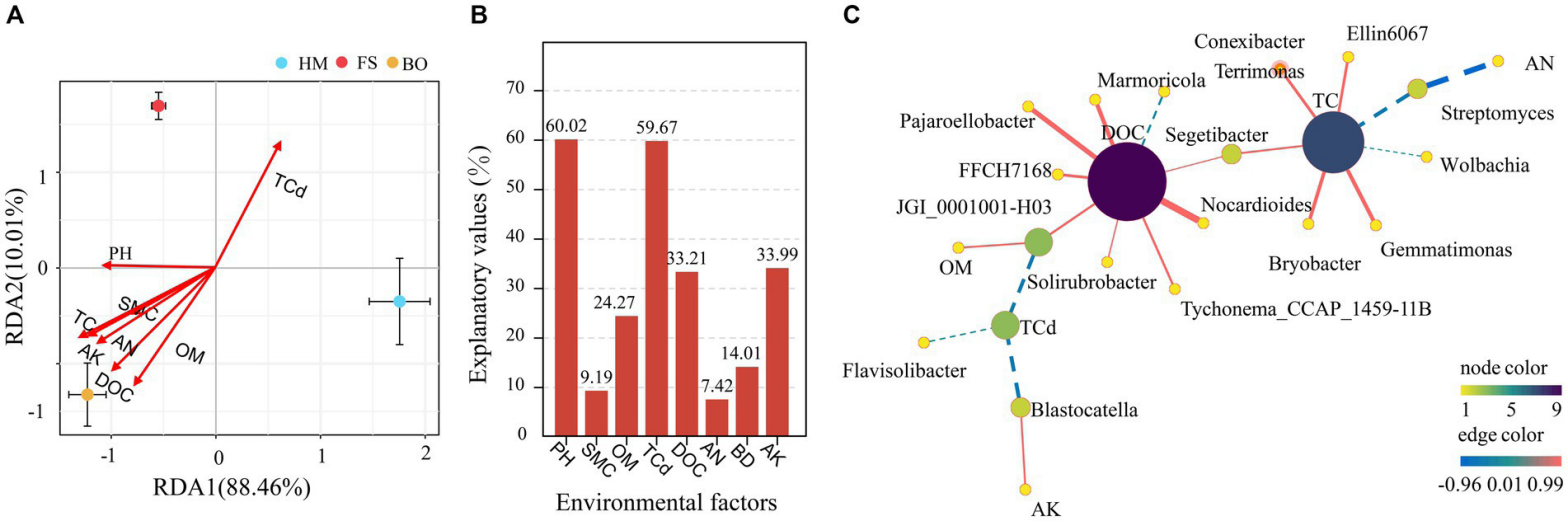
The influence of environmental factors on the distribution of *Sophora* rhizosphere bacterial communities **(A)** redundancy analysis; **(B)** variance partitioning analysis based on OTU; **(C)** genus-level bacterial network analysis.

Furthermore, redundancy analysis (RDA) was conducted to elucidate the associations between the dominant bacterial groups and various environmental factors, including pH, SMC, TC, TCd, DOC, AN, BD, and AK. The environmental variables accounted for 98.47% of the variation in OTU abundance in the bacterial communities. Except for SMC and BD, other environmental factors were significantly correlated with changes in bacteria. Soil TCd, DOC, and TC were the main driving factors for bacterial community diversity. TCd was negatively correlated with pH. These findings indicated that the alterations in soil physicochemical properties significantly influenced the composition of soil bacterial communities (Figure 7A). In addition, to analyze the impact of each environmental factor on microbial community changes, we conducted a variance partitioning analysis (VPA) (Figure 7B). The ranking of environmental factors based on their contribution from large to small is as follows: pH > TCd > DOC > BD > AK > TC > AN > SMC (Figure 7B).

### 3.9 Bacterial network analysis

The study also conducted network analysis based on environmental factors and species. As shown in Figure 7C, all environmental factors had an impact on the bacterial network, except for pH, SMC, and BD. The analysis revealed noteworthy correlations between DOC and eight species, with one exhibiting a negative correlation (*Terrimonas*) and seven displaying positive correlations (*Marmoricola*, *Pajaroellobacter*, *FFCH7168*, *JGI_0001001-H03*, *Solirubtobacter*, *Tychonema_CCAP_145911B*, and *Segetibacter*). TC exhibited significant correlations with seven dominant species, with two showing negative correlations (Wolbachia and Streptomyces) and five showing positive correlations (*Segetibacter*, *Ellin6067*, *Bryobacter*, *Gemmatimonas*, and *Conexibacter*). Furthermore, significant correlations were observed between TCd and three dominant species (*Solirubtobacter*, *Flavisolibacter*, and *Blastocatella*), all of which exhibited negative correlations. A negative correlation was also detected between OM content and *JGI_0001001-H03*. A negative correlation was also observed between AN and *Streptomyces*. Additionally, AK showed a positive correlation with *Blastocatella*.

## 4 Discussion

### 4.1 Effects of the mixed application of biochar and organic fertilizer on soil physicochemical properties and soil enzyme activities

Biochar, an alkaline soil amendment that contains abundant alkaline groups, could facilitate the transformation of dissociated Cd to non-free states, including Cd(OH)_2_, Cd_3_(PO_4_)_2_, and CdCO_3_ (Liu et al., 2011; Udosen et al., 2016; Lee et al., 2022). Recent research has shown that biochar is a cost-effective and environmentally friendly additive for stabilizing and remediating heavy metal-contaminated soils (Yang L. et al., 2020; Yang Y. W. et al., 2020; Sun et al., 2022). A mixture of biochar and organic fertilizer can effectively address issues of soil heavy metal pollution, nutrient imbalance, and growth inhibition of *Mentha crispa* L. (Jiang et al., 2022; Ghassemi and Farhangi, 2023), purslane (Han et al., 2022), alfalfa (Kareem et al., 2022), and rapeseed (Zhang C. et al., 2023; Zhang N. H. et al., 2023; Zhang X. et al., 2023; Zhang Z. et al., 2023; Zhang K. et al., 2023). We also found that the mixed application of biochar and organic fertilizer reduced both TCd in *Sophora* fields and Cd content in *Sophora* (Figures 1A,H; Table 3). Based on previous research, these outcomes could be attributed to two underlying factors. First, biochar has a notable adsorption capacity for Cd (Lian et al., 2015; Muhammad et al., 2018). Second, incorporating biochar-organic fertilizer could increase the soil pH and organic functional group content, facilitating Cd precipitation (Tan et al., 2015; Li et al., 2017; Muhammad et al., 2018, 2019; Zhu et al., 2020; Li et al., 2022). We also found that mixed fertilization had a significant impact on soil physicochemical properties and nutrient levels (Figures 1C–G). These could be ascribed to the direct release of nutrients from organic fertilizers and biochar (Hussain et al., 2023). Furthermore, the improvements in available soil nutrients could be attributed to the high alkalinity and cation exchange capacity (CEC) of biochar (Muhammad et al., 2018; Joshi et al., 2023; Xiao et al., 2023). Nevertheless, it is important to note that although BO treatment mitigated Cd accumulation in *Sophora* tissues, complete avoidance of Cd absorption was not achieved (Table 3).

Typically, the activities of diverse soil enzymes, which are crucial for facilitating the circulation of matter and energy in the soil, are valuable indicators of soil health (Yao et al., 2018; Chow and Pan, 2020; Yan et al., 2022; Joshi et al., 2023). Compared with the HM and FS groups, BO treatment significantly induced the activities of multiple soil enzymes, including catalase, polyphenol oxidase, and β-glucosidase (Table 1). Similar experimental results were also found in other reports (Zheng et al., 2016).

### 4.2 Effects of the mixed application of biochar and organic fertilizer on the growth, Cd content, and matrine and oxymatrine content of *Sophora*

The combined use of biochar and other soil nutrient amendments has been proven to effectively reduce the harmful effects of Cd pollution on plant growth and improve crop productivity (Lian et al., 2015; Pankaj et al., 2016; Liang et al., 2023; Sifton et al., 2023). In our study, we also observed a similar result. BO treatment significantly reduced the Cd content in *Sophora*. *The* roots and rhizomes of *Sophora*, which contain the main active ingredients (matrine and oxymatrine), are used in traditional Chinese medicine. Consequently, the main root length, dry root biomass, matrine, and oxymatrine contents, which are the main indicators of *Sophora* productivity and quality, were significantly improved after the mixed fertilization (Table 2), indicating that this co-application could effectively alleviate the inhibitory effect of Cd on the growth of *Sophora*. Further analysis indicated that changes in soil physicochemical properties and improvements in nutrient balance were mainly responsible for these increases. Similar conclusions have also been found in other studies (Lian et al., 2015; Sifton et al., 2023).

### 4.3 Mixed fertilization may have increased the productivity and quality of *Sophora* plants by regulating the microorganisms in Cd-contaminated soils

Soil bacterial communities are crucial for nutrient cycling, waste decomposition, and pollutant degradation (Muhammad et al., 2019). The presence of heavy metals not only has adverse effects on the physical and chemical properties of soils but can also change the composition, activity, and function of soil bacterial communities (Delgado, 2019; Chao et al., 2023). In turn, the analysis of bacterial community composition can be used to evaluate the effectiveness of the remediation of Cd-contaminated soil (Hussain et al., 2023; Yan et al., 2023).

Our findings suggested that the cultivation of *Sophora* plants with the application of a biochar-organic fertilizer mixture significantly increased the diversity of bacterial communities in Cd-contaminated soil (Figures 5C, 6B), as reported previously (Chow and Pan, 2020; Haider et al., 2021). This effect could be at least partially attributed to the high nutrient content in the biochar-organic fertilizer mixture (Haider et al., 2021). Specifically, supplementing the carbon pool fosters a conducive microenvironment for the proliferation and metabolic processes of soil bacteria, thereby augmenting the biomass and diversity of soil bacteria (Haider et al., 2021; Zheng K. et al., 2023).

Numerous studies have shown a significant correlation between the decrease in soil pH and the systematic aggregation of Acidobacteria enhancement (Conradie and Jacobs, 2021; Xiao et al., 2023). In the HM group, Acidobacteria, including Subgroup_6 in the Acidobacteria taxonomic group, were significantly enriched (Figure 6B). After BO treatment, the relative abundance of Acidobacteria and Subgroup_6 significantly decreased (Figure 6B). This indicates a significant negative correlation between pH increase and the relative abundance of Acidobacteria (Supplementary Figure S1). This change should be caused by the high alkalinity of the biochar-organic fertilizer. In addition, Acidobacteria and Subgroup_6 were found to have oligotrophic growth characteristics in some studies (Anwar et al., 2013; Zheng et al., 2016). Thus, the increase in soil nutrients induced by the BO treatment should also be the reason for the negative correlation mentioned above. In the remediation of soils contaminated with polycyclic aromatic hydrocarbons and heavy metals, it was found that the relative abundance increase of Proteobacteria would lead to better remediation effects (Kuppusamy et al., 2016). Under nutrient-rich conditions, Proteobacteria usually dominate among bacterial species, with a relatively high relative abundance (Amin, 2023). However, sometimes there are differences (Amin, 2023). In the case of remediation of heavy metal-contaminated soils using microbial inoculants and legumes, the increase in AN had a significant impact on the reduction of Proteobacteria, resulting in a decrease in soil TCd and Cd content in *Robinia pseudoacacia* L. (Zheng K. et al., 2023). In our study, the relative abundance of Proteobacteria in the BO group was also negatively correlated with the AN (Figure 6B; Supplementary Figure S1). Additionally, the reduction in Proteobacteria community abundance was beneficial for the decrease in Cd content, longitudinal growth of the main root, and accumulation of dry root biomass and oxymatrine of *Sophora* (Supplementary Figure S3). Although the relative abundance of Bacteroidetes was significantly increased in the BO treatment, it was not significantly correlated with the indicators of soil physicochemical properties (Figure 5C). This may be related to the change in soil aggregate size caused by BO (Fan et al., 2021; Zhang C. et al., 2023; Zhang N. H. et al., 2023; Zhang X. et al., 2023; Zhang Z. et al., 2023; Zheng K. et al., 2023). However, the increase in the relative abundance of Bacteroidetes was beneficial for the decrease in Cd content, longitudinal growth of the main root, and accumulation of dry root biomass and oxymatrine in *Sophora* (Supplementary Figure S3). Similar results were also found in other reports, as Bacteroidetes were determined to be Cd-tolerant microorganisms with positive impacts on plant growth (Lu et al., 2020; Xie et al., 2021; Pfendler et al., 2023). Compared with those in the HM and FS groups, the relative abundance of Firmicutes in the BO group, including *Bacillus* and *Bacillus_acidiceler*, was significantly decreased (Figures 5, 6). As is well known, Firmicutes, including their taxonomic groups, have a higher relative abundance in various extreme environments (Venkateswar and Venkata, 2012; Mukhia et al., 2022; Zuo et al., 2023). The decrease in the relative abundance of Firmicutes was thought to be beneficial for restoring the health of heavy metal-contaminated soils and reducing heavy metal accumulation in crops (Hui et al., 2020; Alsamhary, 2022; Tan et al., 2023; Yang et al., 2023). In addition, Firmicutes, *Bacillus*, and *Bacillus_acidiceler* were positively correlated with TCd and BD, while negatively correlated with pH, OM, DOC, and AK in our study (Supplementary Figures S1, S2). The decrease in the relative abundance of Firmicutes and *Bacillus* promoted the main root length, dry root biomass, and oxymatrine content of *Sophora*, which was beneficial for reducing Cd accumulation in *Sophora* (Supplementary Figures S3, S4). Moreover, the relative abundance of Chloroflexi increased, which was beneficial for the accumulation of main root length, dry root biomass, oxymatrine, and matrine of *Sophora*, as well as for the reduction of Cd content in roots and stems of *Sophora* (Supplementary Figures S3, S4). DOC, pH, and AK are positively correlated with the relative abundance of Chloroflexi, while TCd and BD are significantly negatively correlated with Chloroflexi (Supplementary Figure S1). A negative correlation between Chloroflexi abundance and soil Cd content was also demonstrated (Gondek et al., 2023). In addition to Chloroflexi, Gemmatimonadetes, and Patescibacteria were also significantly enriched in the BO treatment, which was beneficial for the reduction of TCd. Interestingly, among all microbial species, only the proliferation of Chloroflexi and Armatimonadetes could promote the accumulation of matrine (Supplementary Figure S3). Whether these two microbial species could directly or indirectly regulate the biosynthetic pathway of matrine in *Sophora* or even be endophytic bacteria that produce matrine together with *Sophora* needs further investigation.

**Figure 8:**
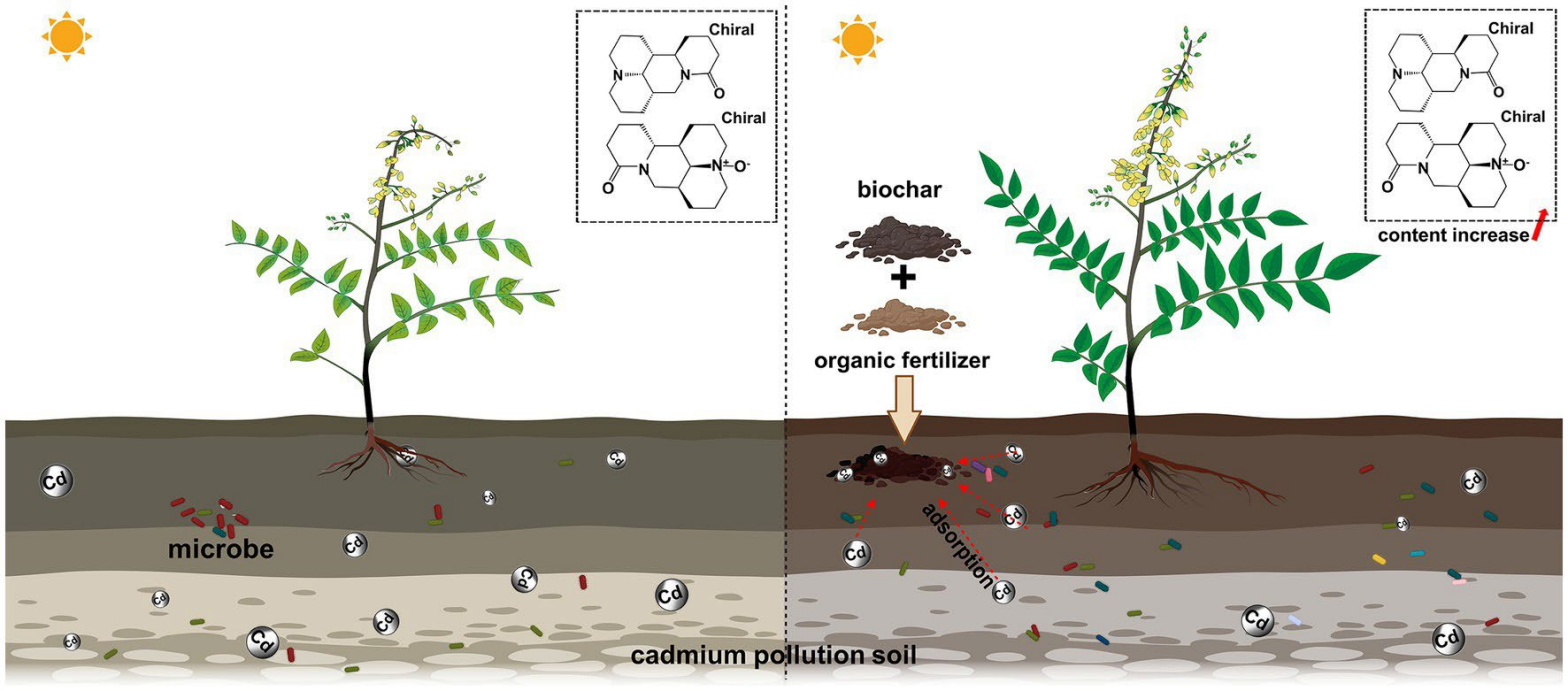
BO treatment changed the health status of the *Sophora* rhizosphere soil and facilitated *Sophora* growth.

In conclusion, the results showed that the combined use of biochar and organic fertilizer in Cd-contaminated *Sophora* fields significantly improved soil health, reduced Cd accumulation, and increased *Sophora* yield and quality (Figure 8). This beneficial effect could be attributed to the changes in the soil’s physicochemical properties, such as pH and soil carbon pool, which were important in regulating the dominant bacterial species or the entire bacterial network. In the future, it will be important to illustrate the proportion of different forms of Cd in soil, the underlying molecular mechanism of Cd absorption by BO treatment, the targeted cultivation of excellent microorganisms in Cd-contaminated soil, and the regulation mechanism of yield and quality of *Sophora* by rhizosphere microorganisms to balance soil health, plant growth, and ecological restoration.

## 5 Conclusion

The potential value of the mixed application of biochar and organic fertilizer to *Sophora* in Cd-contaminated soil was evaluated from three aspects: soil properties, crop yield, and bacterial structure. This fertilization method created a richer and more diverse rhizosphere microenvironment, achieving higher *Sophora* yield and higher levels of major secondary metabolites. In addition, the biochar-organic fertilizer mixture had a significant impact on microbial communities, including Acidobacteria, Proteobacteria, Bacteroidetes, Firmicutes, Chloroflexi, Gemmatimonadetes, Patescibacteria, Armatimonadetes, Subgroups_ 6, *Bacillus* and *Bacillus_ Acidiceler*. The relative changes of these microbial communities are influenced by soil physicochemical properties and can potentially improve the productivity and quality of *Sophora*. Overall, in terms of improving the health of the Cd-contaminated soils and increasing the yield and quality of *Sophora*, this new approach is relatively effective and desirable.

## Data availability statement

The datasets presented in this study can be found in online repositories. The names of the repository/repositories and accession number(s) can be found in the article/Supplementary material.

## Author contributions

HL: Data curation, Formal analysis, Investigation, Methodology, Resources, Writing – original draft. CL: Formal analysis, Investigation, Methodology, Resources, Writing – original draft. YL: Resources, Validation, Writing – original draft. Y-jC: Resources, Validation, Writing – original draft. Z-jZ: Resources, Validation, Writing – review & editing. K-hW: Conceptualization, Writing – review & editing. ML: Conceptualization, Data curation, Methodology, Writing – review & editing.

## Footnotes

^1^ http://www.omicsmart.com
