# Correction: Biochar and organic fertilizer drive the bacterial community to improve the productivity and quality of *Sophora tonkinensis* in cadmium-contaminated soil

**DOI:** 10.3389/fmicb.2026.1784094

**Published:** 2026-01-27

**Authors:** Han Liu, Cui Li, Yang Lin, Yi-jian Chen, Zhan-jiang Zhang, Kun-hua Wei, Ming Lei

**Affiliations:** 1 National Center for TCM Inheritance and Innovation, Guangxi Botanical Garden of Medicinal Plants, Nanning, China; 2 Guangxi Key Laboratory of Medicinal Resources Protection and Genetic Improvement, Guangxi Botanical Garden of Medicinal Plants, Nanning, China; 3 Guangxi Engineering Research Center of TCM Resource Intelligent Creation, Guangxi Botanical Garden of Medicinal Plants, Nanning, China; 4 The Third Affiliated Hospital of Heilongjiang University of Traditional Chinese Medicine, Harbin, China; 5 Guangxi Key Laboratory for High-Quality Formation and Utilization of Dao-di Herbs, Guangxi Botanical Garden of Medicinal Plants, Nanning, China

**Keywords:** biochar and organic fertilizer, cadmium-contaminated soil, *Sophora tonkinensis*, bacterial community, yield and quality

The funder the Guangxi Science and Technology Base and Talent Project, [GuikeAD22080016] to Ming Lei was erroneously omitted.

The updated Funding statement is below:

The author(s) declared that financial support was received for this work and/or its publication. This research was funded by the Guangxi Science and Technology Base and Talent Project (Guike AD22080016), the National Natural Science Foundation of China (82260747); the Natural Science Foundation of Guangxi (2020GXNSFAA159151); Central Guiding Local Science and Technology Development Special Project (Guike ZY20198018); Guangxi Elite Team of Medicinal Plant Conservation (009699); and Key Techniques Research and Promotion of Guangxi Medicinal Materials Varieties (GZKJ2314).

The original version of this article has been updated.

