# Supplementary material for: Biochar and organic fertilizer drive the bacterial community to improve the productivity and quality of Sophora tonkinensis in cadmium-contaminated soil
Source: Front Microbiol. 2024 Jan 8;14:1334338. doi: 10.3389/fmicb.2023.1334338 (PMC10800516; doi:10.3389/fmicb.2023.1334338)
Supplement: Supplementary file 3 [file Table_3.DOCX]

Supplementary Material

Biochar and organic fertilizer drives the bacterial community to improve the productivy and quality of *Sophora tonkinensis* in cadmium contaminatd soil

Han Liu^1,2,3†^, Cui Li^1,2,3†^, Yang Lin^1,2,3^, Yi-jian Chen^4^, Zhan-jiang Zhang^1,5^, Kun-hua Wei^1,2,3*^, Ming Lei^1,2,3*^,r

*** Correspondence:**Kun-hua Wei
[divinekh@163.com](mailto:divinekh@163.com)

Ming Lei
leiming@gxyyzwy.com

# Supplementary Figures


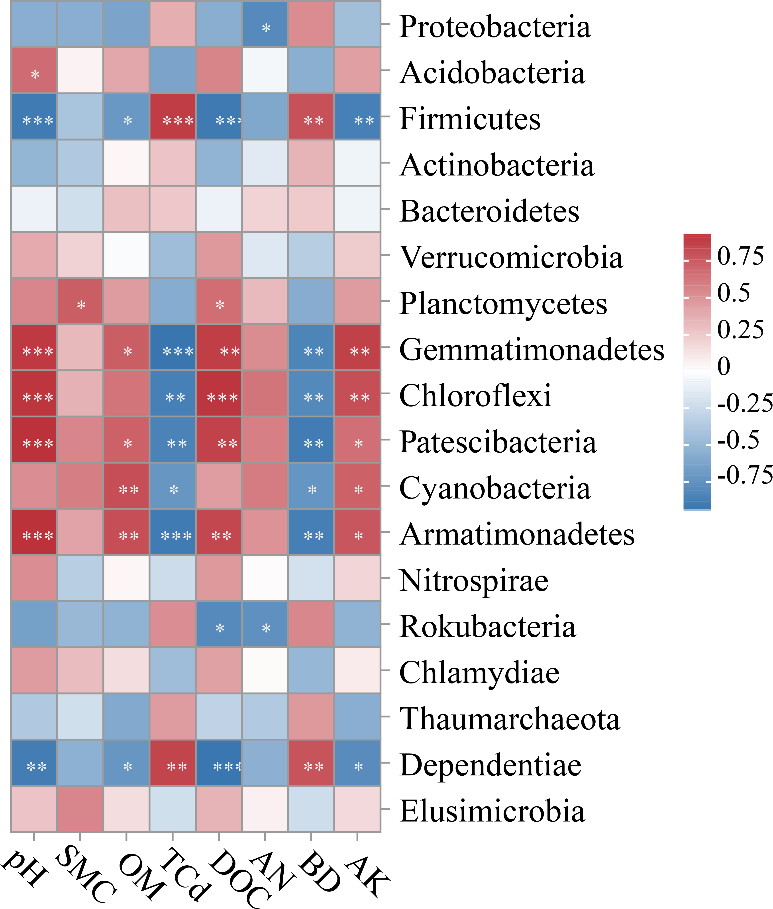


**Supplementary Figure 1.** The correlations between soil properties and the relative abundance of bacterial communities at the phylum level. pH: Soil pH; SMC: soil moisture content; OM: soil organic matter content; TCd: soil total cadmium content; DOC: soil dissolved organic carbon content; AN: soil alkali-N content; BD: soil bulk density content; AK: soil available potassium. Red represents positive correlation, blue representing negative correlation, the darker the color, the stronger the correlation. The symbol “*” indicates statistical significance at p < 0.05, “**” indicating p < 0.01, and “***” indicating p < 0.001.


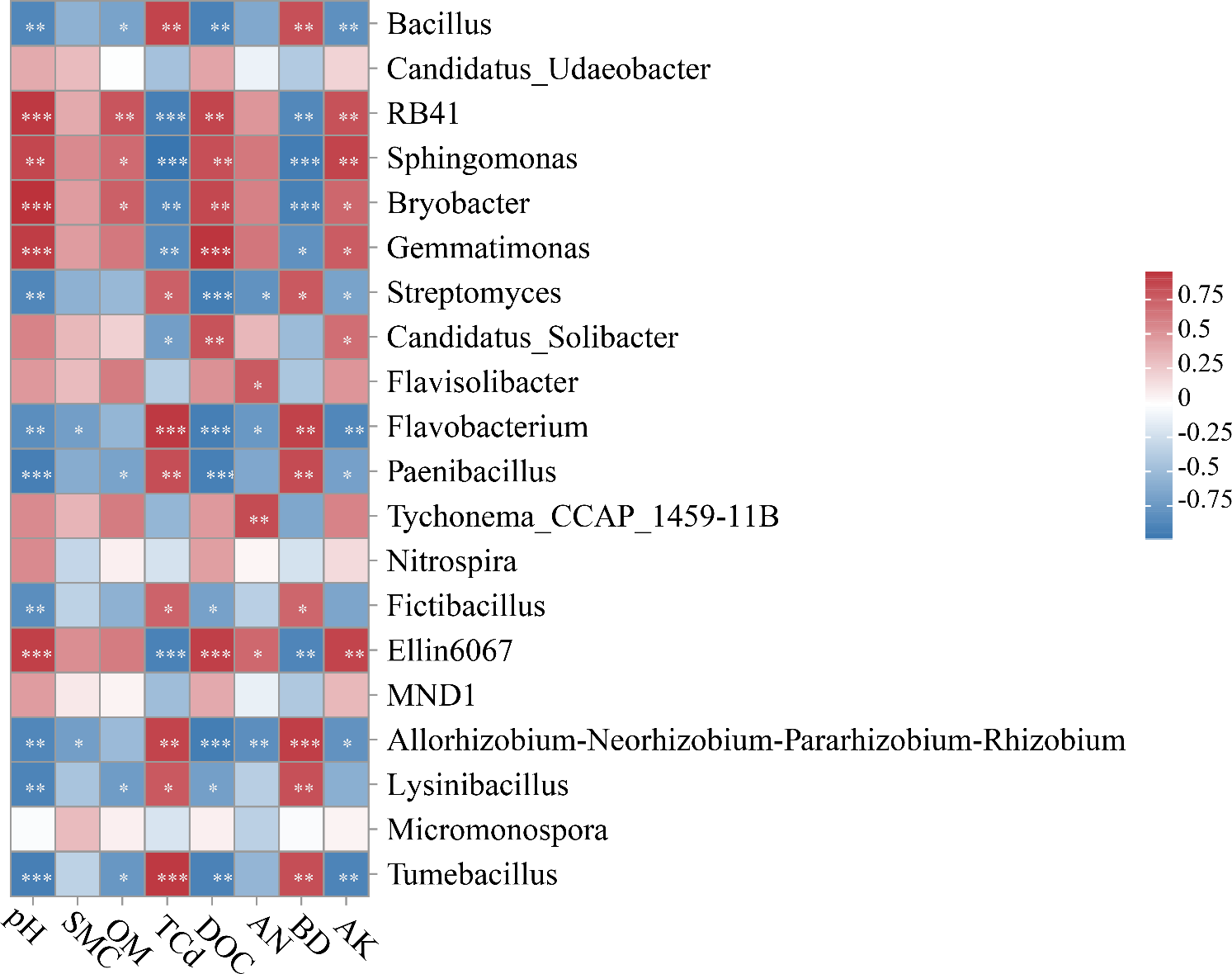


**Supplementary Figure 2.** The correlations between soil properties and the relative abundance of bacterial communities at the genus level. Red represents positive correlation, blue representing negative correlation, the darker the color, the stronger the correlation. The symbol “*” indicates statistical significance at p < 0.05, “**” indicating p < 0.01, and “***” indicating p < 0.001.


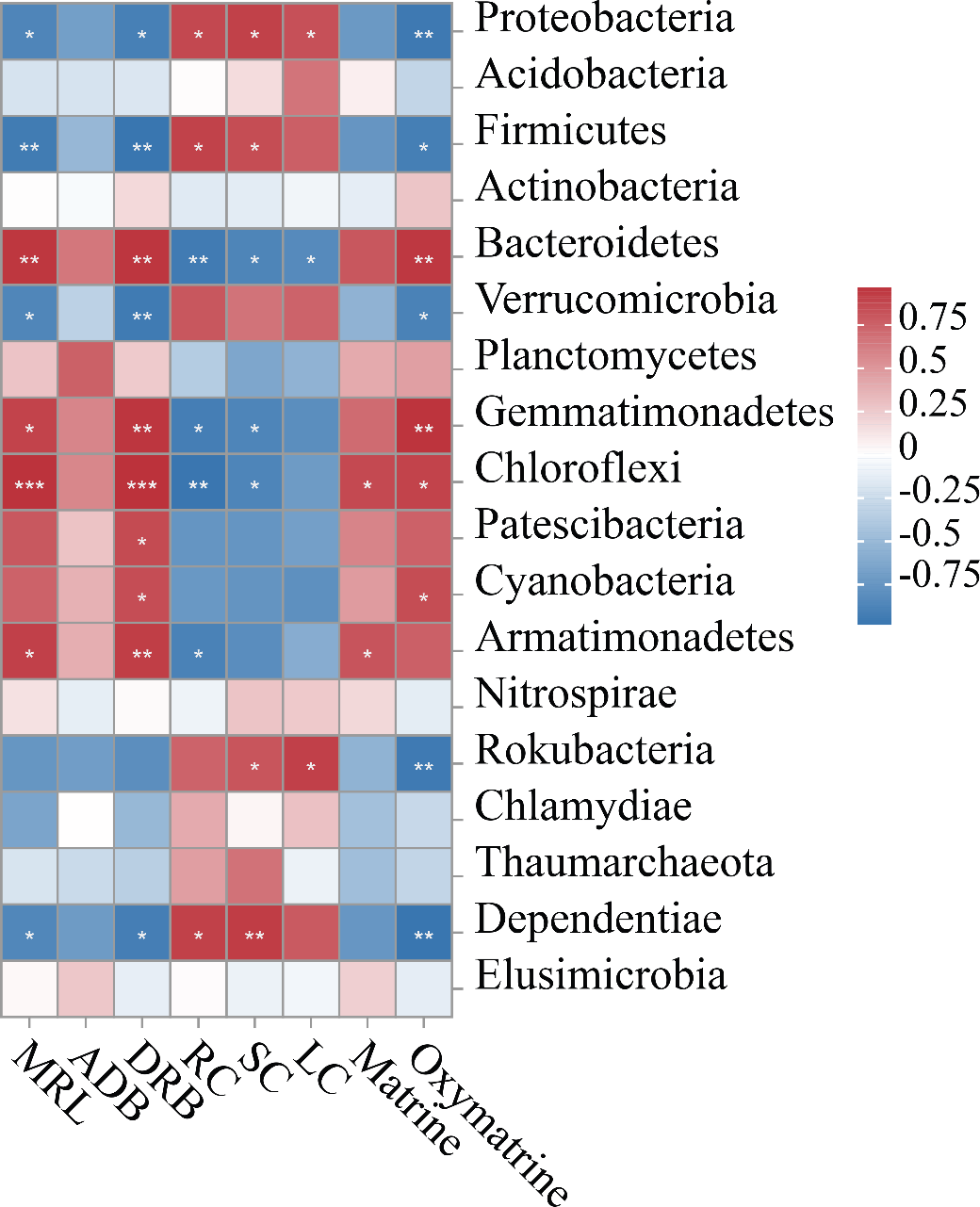


**Supplementary Figure 3.** The correlations between the relative abundance of bacterial communities at the phylum level and the productivity and quality of *Sophora*. MRL: main root length; ADB: aboveground dry biomass; DRB: dry root biomass; RC: root Cd content; SC: stem Cd content; LC: leaf Cd content. Red represents positive correlation, blue representing negative correlation, the darker the color, the stronger the correlation. The symbol “*” indicates statistical significance at p < 0.05, “**” indicating p < 0.01, and “***” indicating p < 0.001.


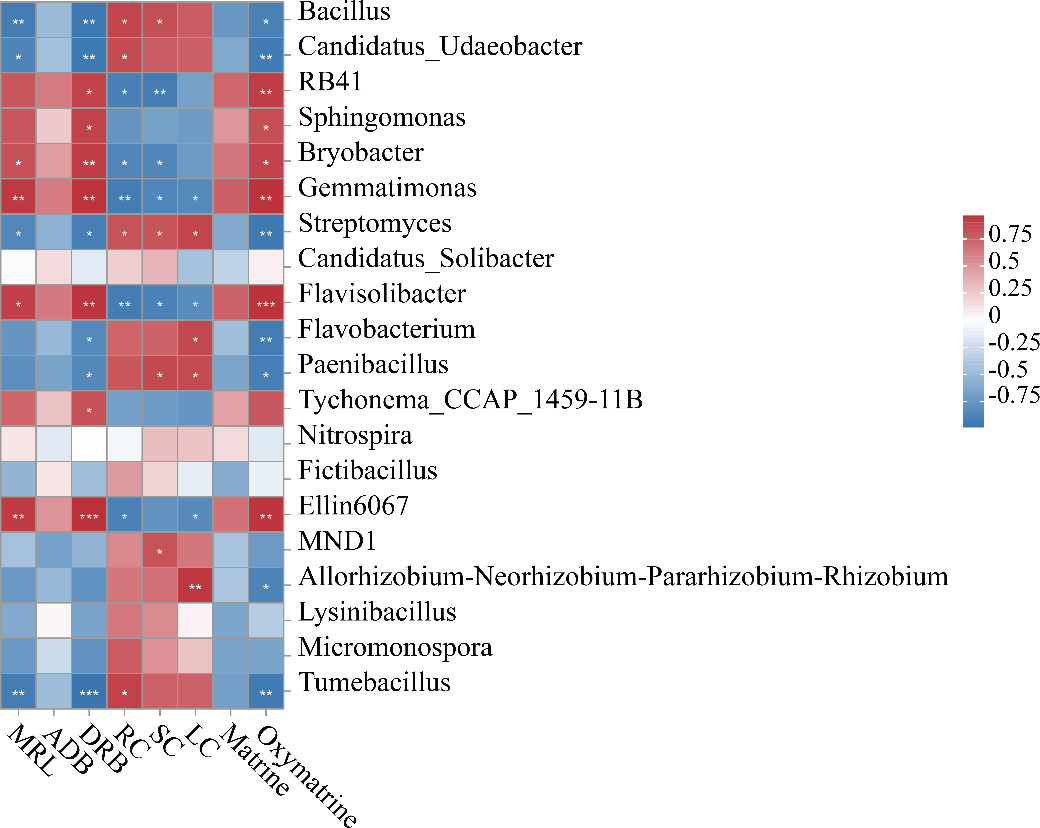


**Supplementary Figure 4.** The correlations between the relative abundance of bacterial communities at the genus level and the productivity and quality of *Sophora*. Red represents positive correlation, blue representing negative correlation, the darker the color, the stronger the correlation. The symbol “*” indicates statistical significance at p < 0.05, “**” indicating p < 0.01, and “***” indicating p < 0.001.
