# Supplementary figures and images for: Biochar and organic fertilizer drive the bacterial community to improve the productivity and quality of Sophora tonkinensis in cadmium-contaminated soil
Source: Front Microbiol. 2024 Jan 8;14:1334338. doi: 10.3389/fmicb.2023.1334338 (PMC10800516; doi:10.3389/fmicb.2023.1334338)

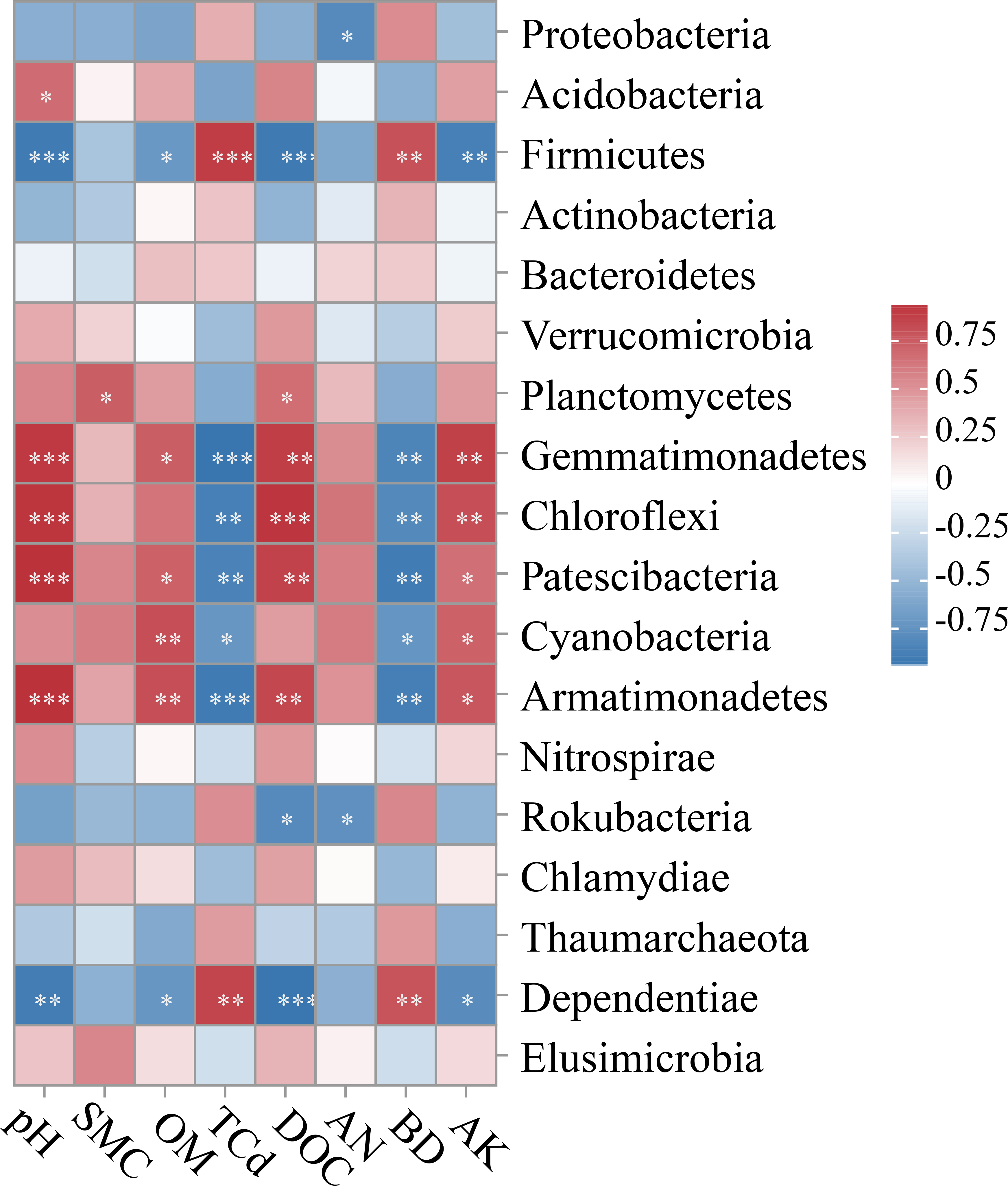

Supplement: Supplementary file 4 [file Image_1.TIF]

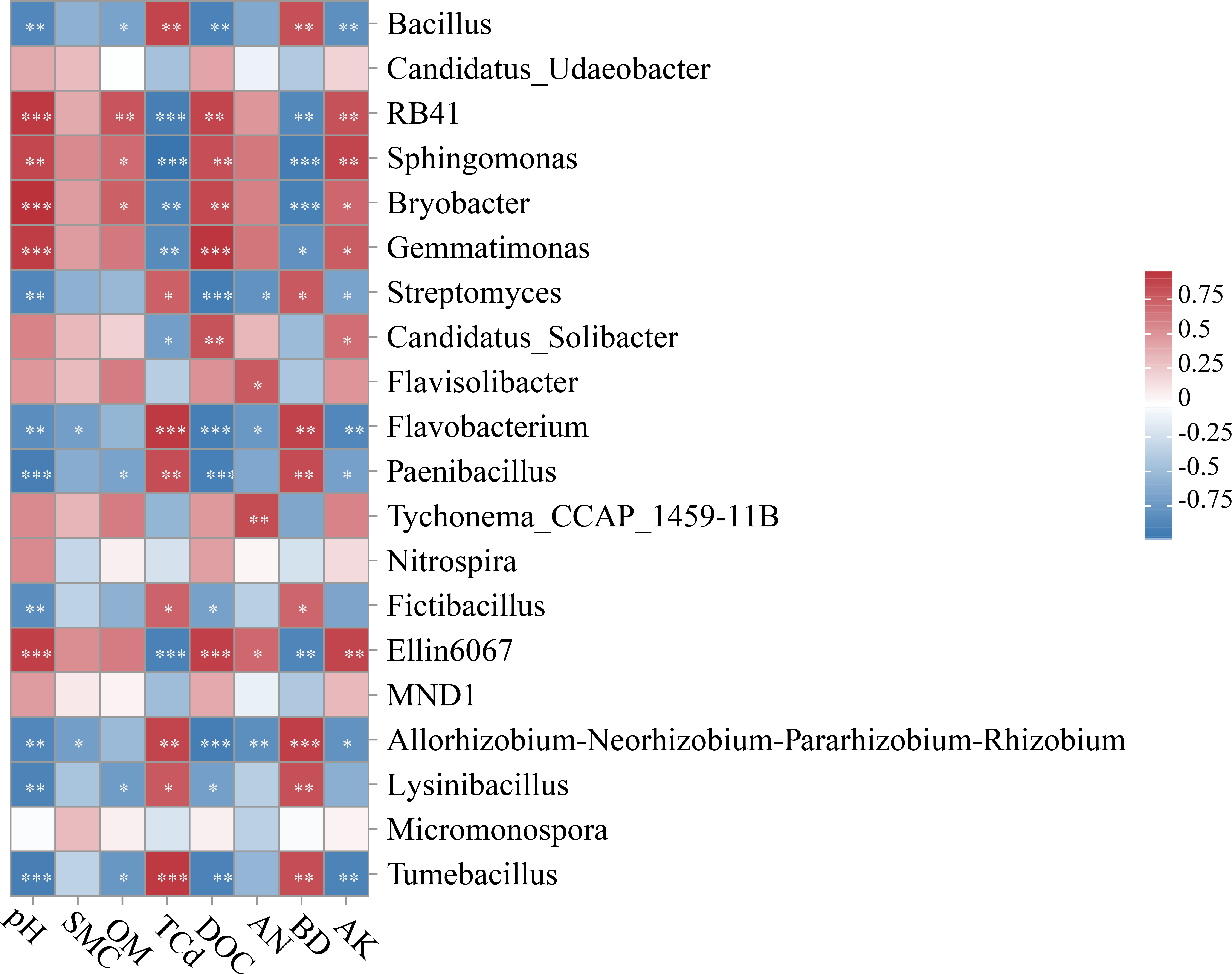

Supplement: Supplementary file 5 [file Image_2.TIF]

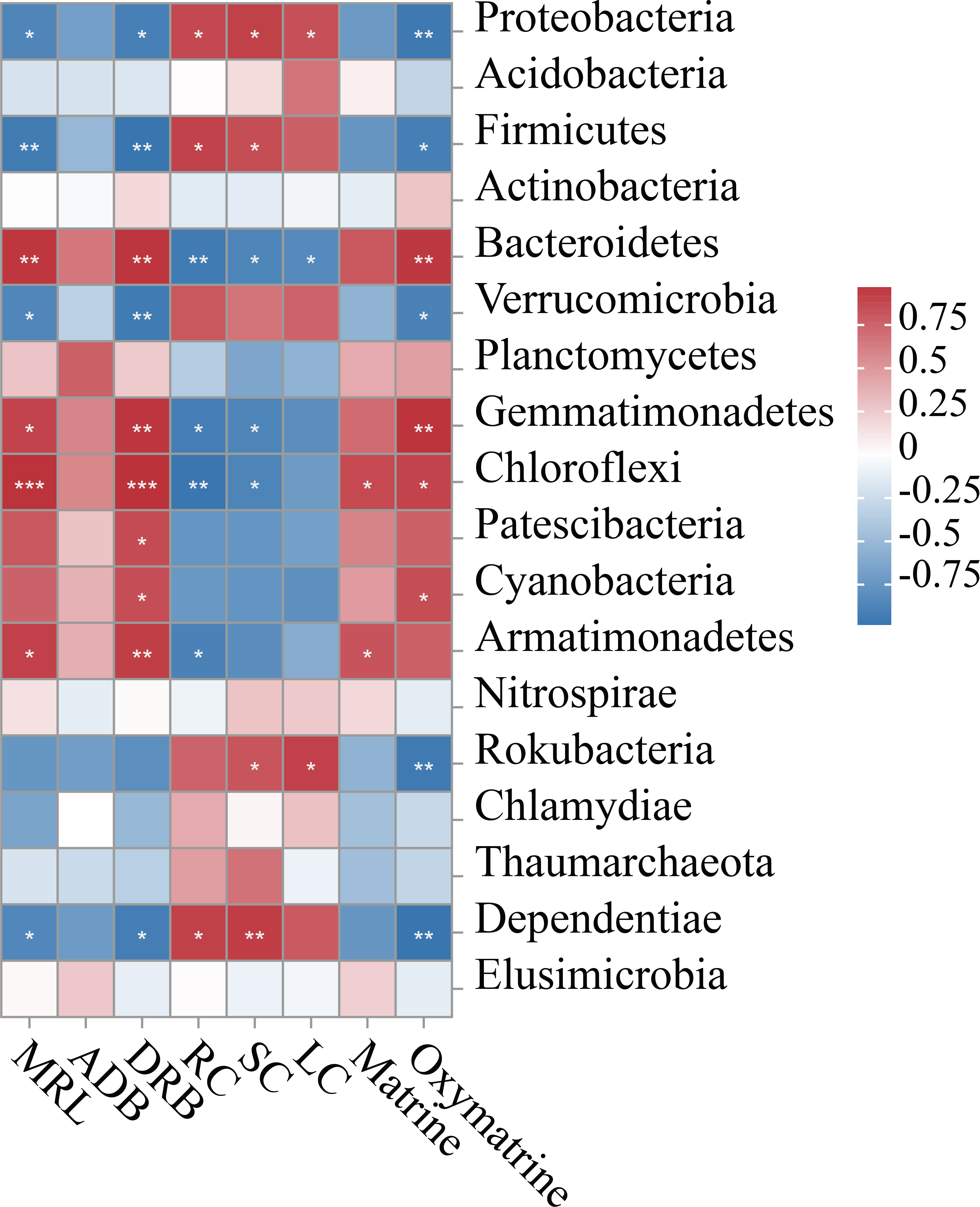

Supplement: Supplementary file 6 [file Image_3.TIF]

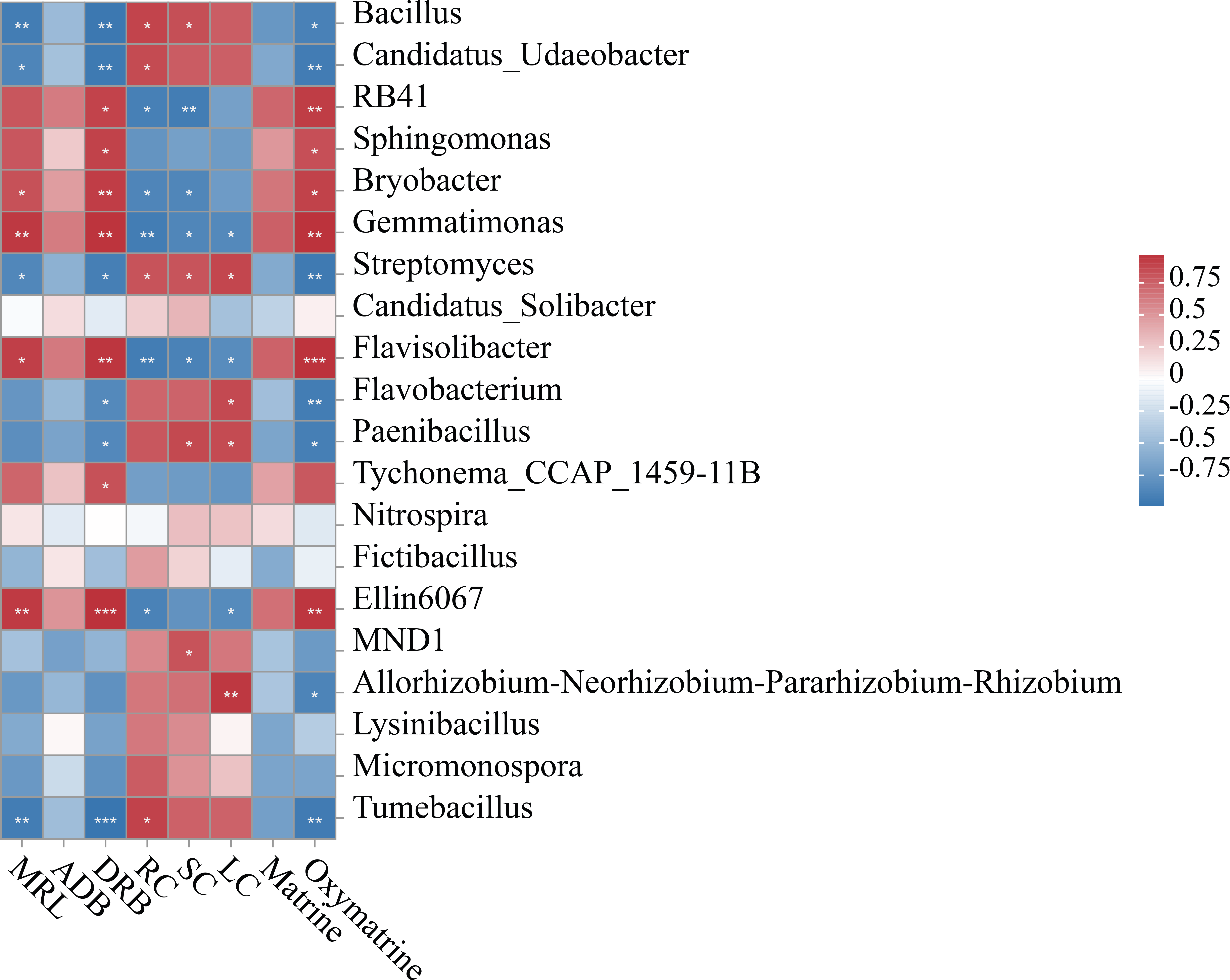

Supplement: Supplementary file 7 [file Image_4.TIF]
